# Association of Somatic Symptom Severity With Sociodemographic Parameters In Patients With Medically Unexplained Physical Symptoms: A Cross-Sectional Study From a Tertiary Care Center in India

**DOI:** 10.7759/cureus.9250

**Published:** 2020-07-17

**Authors:** Upendra Baitha, Piyush Ranjan, Koushik Sinha Deb, Nitesh K Bauddh, Vishwajeet Singh, Gaurishanker Kaloiya, Arvind Kumar, Anamika Sahu

**Affiliations:** 1 Medicine, All India Institute of Medical Sciences, Delhi, IND; 2 Psychiatry, All India Institute of Medical Sciences, Delhi, IND; 3 Medicine, Government Medical College, Kota, IND; 4 Biostatistics, All India Institute of Medical Sciences, Delhi, IND; 5 Clinical Psychology, All India Institute of Medical Sciences, Delhi, IND

**Keywords:** medically unexplained physical symptoms, somatic symptoms, correlates, india

## Abstract

Background

There is a paucity of studies assessing the severity of somatic symptoms in medically unexplained physical symptoms (MUPS) from Medicine outpatient department (OPD).

Methodology

This cross-sectional study was conducted in Medicine OPD of a tertiary care hospital in India, in which 245 MUPS-diagnosed patients out of 976 consecutive screened patients were evaluated for the severity of somatic symptoms (by administering the Patient Health *Questionnaire*-15) and its sociodemographic correlates.

Results

Out of 245 recruited patients, three-fourth had a significant severity level of somatic symptoms. High level of somatic symptom severity was more common in females (p ≤ 0.001), married patients (p = 0.011), rural dwellers (p = 0.035), less educated (p = 0.003), and those with lower socioeconomic status (p = 0.001).

Conclusions

Patients with MUPS have a high level of somatic symptom severity with certain sociodemographic correlates. Further research should be conducted to investigate the reasons for this and to formulate a cost-effective treatment strategy.

## Introduction

Patients with medically unexplained physical symptoms (MUPS) are characterized by persistent somatic complaints for which adequate clinical examination and relevant investigations do not reveal sufficient explanation by known disease. These patients present with multiple somatic symptoms such as pain, fatigue, functional disturbances, and other systemic complaints such as neurological and gastrointestinal complaints.

They constitute a considerable portion of patients in all levels of health care, particularly in general care settings where prevalence rates are reported between 20% and 30% [1,2]. Typically, these patients seek multiple consultations from different specialties and utilize more health care services, leading to a substantial burden on health care systems [3]. Studies suggest that these patients have significant disabilities and poor quality of life, which may be due to the high burden of somatic symptoms [4,5].

Studies assessing the severity of somatic symptoms in patients with MUPS from the Medical outpatient department (OPD) facilities are scarce. One such study with a small sample size suggests that the severity of somatic symptoms is more in patients with MUPS than in patients with medically explained symptoms [6]. The sociodemographic correlates of the severity of somatic symptoms in MUPS have not been studied elaborately. We conducted this cross-sectional study to assess the severity of somatic symptoms by administering the Patient Health Questionnaire-15 (PHQ-15) among patients with MUPS in a tertiary care hospital of India and evaluated the sociodemographic factors affecting the severity of the symptoms in these patients.

## Materials and methods

This cross-sectional observational study was conducted in the Medicine OPD of a tertiary care hospital in North India. The Institution Ethics Committee approved the study, and written consent was taken from all patients recruited in the study. A total of 976 consecutive patients presenting to the designated OPD rooms of Medicine department, over a period of one year were assessed for MUPS and were recruited according to the inclusion and exclusion criteria. For this study, MUPS was defined as any current principal symptom(s) in a patient for which no medical diagnosis could be reached by detailed clinical examination and appropriate investigations [7]. Each case was reviewed by two independent physicians before establishing a diagnosis of MUPS. Referrals from other subspecialties were also taken. Patient's data regarding sociodemographic correlates such as age, gender, marital status, educational level, employment status, and occupation were recorded in pre-validated preform that was developed specifically for the index study. Assessment of the severity of the symptoms was performed using PHQ-15 [8,9]. PHQ-15 is a validated 15-item questionnaire that assesses the severity of somatic symptoms. Each item asks the participant to rate the severity of the somatic symptom experienced during the past seven days on a 3-point scale (0 if not bothered at all, 1 if bothered a little, and 2 if the symptom bothered a lot). The total score varies from 0 to 30, with higher scores signifying greater severity of somatic symptoms. The level of somatic symptom severity is interpreted as minimal, low, moderate, and high for a PHQ-15 score of 0-4, 5-9, 10-14, and 15-30, respectively.

Each qualitative variable was expressed as an absolute and relative frequency, whereas the continuous variable was expressed as mean along with standard deviation. To find the association between qualitative variables, the chi-square test or Fisher exact test was used according to the distribution of data. A p-value of less than 0.05 was considered statistically significant.

## Results

A total of 976 consecutive patients who presented to the designated OPD rooms of the Medicine Department were assessed for the presence of MUPS. Among them, 245 patients were diagnosed to have MUPS and were subsequently recruited in the study. Most of the patients included in the study were young, married, and living in a joint family. The majority of them belonged to the middle socioeconomic class and have completed school education. Sixty-one (25%) patients had a minimal/low level of severity of somatic symptoms, whereas 105 (46%) and 79 (29%) patients had medium and high levels of severity of somatic symptoms, respectively. Their sociodemographic parameters, levels of severity of somatic symptoms, and an association between the two are included in Table 1.

**Table 1:** Association between symptoms severity and sociodemographic parameters

Variables	Numbers of patients with MUPS				p-Value
	Total (n = 245)	Levels of the severity of symptoms			
		Minimal/low (n = 61)	Medium (n = 105)	High (n = 79)	
Age (in years)					
Less than 30 years	86 (35%)	28 (46%)	37 (35%)	21 (26%)	0.185
30-40 years	91 (37%)	23 (38%)	36 (34%)	32 (40%)	
40-50 years	49 (20%)	06 (10%)	23 (22%)	20 (25%)	
More than 50 years	19 (8%)	04 (6%)	09 (9%)	06 (9%)	
Gender					
Male	107 (44%)	40 (66%)	48 (46%)	19 (24%)	<0.001
Female	138 (56%)	21 (34%)	57 (54%)	60 (76%)	
Marital status					
Married	176 (72%)	34 (56%)	79 (75%)	63 (80%)	0.011
Single	62 (25%)	25 (41%)	24 (23%)	13 (16%)	
Other	7 (3%)	2 (3%)	2 (2%)	3 (4%)	
Locality					
Urban	138 (56%)	41 (67%)	61 (58%)	36 (46%)	0.034
Rural	107 (44%)	20 (33%)	44 (42%)	43 (54%)	
Family type					
Residing	18 (7%)	06 (10%)	09 (9%)	03 (4%)	0.544
Alone	85 (30%)	23 (38%)	3 (31%)	29 (37%)	
Nuclear joint	142 (58%)	32 (52%)	63 (60%)	47 (59%)	
Socioeconomic class					
Upper	22 (09%)	11 (18%)	10 (9%)	01 (1%)	0.001
Upper middle	51 (21%)	17 (28%)	25 (24%)	09 (11%)	
Lower middle	58 (23%)	13 (21%)	26 (25%)	19 (24%)	
Upper lower	95 (39%)	19 (31%)	36 (34%)	40 (51%)	
Lower	19 (8%)	01 (02%)	08 (8%)	10 (13%)	
Education					
Illiterate	57 (23%)	08 (13%)	22 (21%)	26 (33%)	0.003
Primary school	45 (18%)	08 (13%)	18 (17%)	19 (24%)	
Secondary	79 (32%)	19 (31%)	37 (35%)	23 (29%)	
School graduate and above	65 (25%)	26 (43%)	28 (27%)	11 (14%)	

## Discussion

The study of somatic symptom burden is of paramount importance in any disease. It is directly related to the sense of well-being, quality of life, and health care expenses [10,11]. The study of somatic symptom burden has attracted the interests of researchers from different parts of the world and researchers have studied it in common clinical conditions such as anxiety, depression, chronic obstructive pulmonary disease (COPD), and cardiac illnesses [12-14]. In a large study involving 1,329 patients with anxiety and depression disorders, it has been found that 72.2%, 20.7%, and 7.1% of patients had a minimal/mild, medium, and high level of somatic symptom severity, respectively [12]. In another study involving COPD patients, it was found that minimal/mild, medium, and high level of somatic symptom severity was present in 45.5%, 27.3%, and 27.3% of the patients studied, respectively [13]. In comparison to these common conditions, patients with MUPS have a notably high level of somatic symptom severity. Three-fourth of the consecutive patients of MUPS enrolled in our study had significant (medium or high) levels of the severity of somatic symptoms, of which one-third of the patients had high levels of somatic symptom severity. These findings are in accordance with a cross-sectional retrospective study conducted on 294 primary care patients with MUPS in Germany, which revealed that 29%, 37%, and 34% of the MUPS patients had low, medium, and high levels of somatic symptom severity, respectively [3].

The sociodemographic evaluation of the findings of the index study suggests that a high level of somatic symptom severity is associated with female gender (p < 0.001) and married status (p = 0.011). This finding can be attributed to women’s tendency to perceive physical sensations as more bothersome and seek more frequent medical help in comparison to their male counterparts [15,16]. Women are emotionally more responsive and expressive than men and thus their somatic complaints are more likely to be evaluated and reported [17]. It has been reported that married and widowed women are at higher risk of developing MUPS than single women because of the emotional and social stresses that these women are subjected to, and these emotions get expressed through physical symptoms that are acceptable to the society [18]. Besides, incidences of past abuses and trauma are more common in females [19].

In our study, patients with lower socioeconomic status and less education had a more likelihood of developing high levels of somatic symptom severity. Studies have been conducted to establish a relationship between socioeconomic status, education status, and common mental disorders. Although most of these studies establish a positive correlation between lower socioeconomic status and poor mental health outcomes, some have reported contrary findings [20-22]. Education is supposed to enhance people’s skills and empower them with a better coping mechanism, leading to better mental health [23].

This study is one of the initial large studies that evaluated levels of severity of somatic symptoms in patients with MUPS. MUPS is a common yet neglected clinical entity in this part of the world. The patients with MUPS have higher somatic symptom severity than many other common clinical conditions and have interesting sociodemographic correlates that have not been studied elaborately yet. This study has found that MUPS patients who are females, married, residing in rural areas, less educated, and with lower socioeconomic status are more likely to have a severe level of somatic symptoms. The prevalence of co-existing physical and psychological co-morbidities and their impact on symptom severity in MUPS patients have not been assessed in this study but can be explored in future research. Also, newer treatment modalities such as yoga can be explored that may be useful in reducing somatic symptom severity, quality of life, and associated psychiatric comorbidities [24]. Multidisciplinary treatment modalities such as an MUPS clinic should be opened in association with psychiatrists and psychologists.

This being a cross-sectional study, the causal association and odds ratio cannot be measured, and this is a limitation of the study. Cohort studies should be conducted to establish a causal association.

## Conclusions

Patients with MUPS have a considerable level of somatic symptom severity. MUPS patients with female gender, married status, rural residence, lower socioeconomic status, and less education level have a high level of somatic symptom severity.
